# BacSPaD: A Robust Bacterial Strains’ Pathogenicity Resource Based on Integrated and Curated Genomic Metadata

**DOI:** 10.3390/pathogens13080672

**Published:** 2024-08-09

**Authors:** Sara Ribeiro, Guillaume Chaumet, Karine Alves, Julien Nourikyan, Lei Shi, Jean-Pierre Lavergne, Ivan Mijakovic, Simon de Bernard, Laurent Buffat

**Affiliations:** 1 AltraBio SAS, 69007 Lyon, Francelaurent.buffat@altrabio.com (L.B.); 2 Bases Moléculaires et Structurales des Systèmes Infectieux, IBCP, Université Lyon 1, CNRS, UMR 5086, 69007 Lyon, France; 3 Division of Systems and Synthetic Biology, Department of Life Sciences, Chalmers University of Technology, 412 96 Göteborg, Sweden; 4 Novo Nordisk Foundation Center for Biosustainability, Technical University of Denmark, 2800 Lyngby, Denmark

**Keywords:** bacterial pathogenicity, genomic metadata analysis, bioinformatics, microbiology research, public health surveillance

## Abstract

The vast array of omics data in microbiology presents significant opportunities for studying bacterial pathogenesis and creating computational tools for predicting pathogenic potential. However, the field lacks a comprehensive, curated resource that catalogs bacterial strains and their ability to cause human infections. Current methods for identifying pathogenicity determinants often introduce biases and miss critical aspects of bacterial pathogenesis. In response to this gap, we introduce BacSPaD (Bacterial Strains’ Pathogenicity Database), a thoroughly curated database focusing on pathogenicity annotations for a wide range of high-quality, complete bacterial genomes. Our rule-based annotation workflow combines metadata from trusted sources with automated keyword matching, extensive manual curation, and detailed literature review. Our analysis classified 5502 genomes as pathogenic to humans (HP) and 490 as non-pathogenic to humans (NHP), encompassing 532 species, 193 genera, and 96 families. Statistical analysis demonstrated a significant but moderate correlation between virulence factors and HP classification, highlighting the complexity of bacterial pathogenicity and the need for ongoing research. This resource is poised to enhance our understanding of bacterial pathogenicity mechanisms and aid in the development of predictive models. To improve accessibility and provide key visualization statistics, we developed a user-friendly web interface.

## 1. Introduction

### 1.1. Leveraging Bacterial Omics Data for Pathogenicity Insights and Public Health

In microbiology research, the vast availability of bacterial omics data is a crucial asset for exploring the diverse aspects of bacterial human pathogens. This wealth of information is instrumental in developing large-scale, in-depth research aimed at expanding our knowledge on omics pathogenicity determinants, critically enhancing public health surveillance and facilitating the development of novel therapeutic strategies. The utilization of bioinformatics to analyze these data has been key in uncovering mechanisms of infection and resistance in established pathogens [1,2,3]. Complete genome data is particularly advantageous for these studies due to the need for high accuracy and completeness in genome assembly [4,5,6]. By integrating this molecular data with epidemiological and clinical information, we can develop a more complete picture of bacterial pathogenesis. Data-driven insights may also contribute to the development of important predictive models to uncover the pathogenic potential of newly identified bacterial strains [7,8,9,10].

### 1.2. Current Challenges

Despite the advances in omics technologies, significant challenges remain in the annotation of bacterial pathogenicity. A primary challenge is managing the vast volumes of data generated, requiring robust analytical methods for accurate classification and interpretation. Indeed, there is currently no publicly accessible and curated database that categorizes bacterial strains based on their human pathogen potential. To construct their training sets, past studies that developed pathogenicity prediction tools classified their selected genomes as pathogenic to humans (HP) and non-pathogenic to humans (NHP) using predominantly two methods.

The first method involved retrieving the information from databases, such as the National Center for Biotechnology Information (NCBI) [11], Genomes Online Database [12], and the Integrated Microbial Genomes database [13]. However, most of these annotations are no longer available. While the exact reasons for this are not explicitly stated in the available literature, concerns about the accuracy of pathogenicity labels and the difficulty in keeping up with the influx of new genomic data are plausible explanations. Other related databases that integrate comprehensive genomic data include BacDive [14], gcPathogen [15], BacWGSTdb [16], and MGTdb [17]. Although these databases provide an extensive and high-quality genomic resource, they lack annotations on bacterial pathogenicity at the strain level and for complete genomes. BacDive provides a limited number of genomes with pathogenicity annotations with unclear criteria. Similarly, gcPathogen, BacWGSTdb, and MGTdb primarily rely on species-level classifications derived from government health organizations. As emphasized in [18], pathogenicity is more accurately assessed at the subspecies level and can vary significantly even within serovars. Incorporating these pathogenicity assessments could then improve the utility of pathogen inventories for research and public health efforts.

The second and most recently used method is the application of an annotation-based pathogenicity labeling, by applying a set of rule-based criteria to genome metadata [7,9,10]. This method is inherently adaptable, and the transparency afforded by the explicit criteria ensures that the process is verifiable. Moreover, its capacity to leverage available metadata broadens its analytical scope, enabling a more comprehensive exploration of bacterial genomes and thereby enriching pathogenicity research. By considering various types of information—such as isolation source, associated disease, sample type, and known interactions with hosts—the method can provide a more nuanced understanding of which bacterial strains are HP or NHP. However, the guiding principle that was generally used in these works to establish the rule-based criteria was that any bacterium isolated from a diseased individual should be considered as HP, while those from healthy individuals or probiotic supplements should be considered NHP. Yet, isolating a bacterium from a diseased individual does not confirm it as the causative agent of the illness [19]. For instance, a bacterium isolated from someone with a non-infectious condition, such as Crohn’s disease, would be wrongly labeled as HP, despite not causing an infectious disease. Similarly, isolating a bacterium from a healthy individual does not automatically indicate that it is NHP. Erroneous assumptions in pathogenicity classification risk introducing bias, potentially leading to the oversight of genes or proteins that are critical for understanding bacterial pathogenicity or for developing prediction tools. Indeed, an automatic method based on keywords was used in the context of these works, which, while useful, may lead to incorrect classifications due to a lack of context interpretation. A commonly used database to retrieve genomic and related data in the field of infectious diseases from previously described studies was PAThosystems Resource Integration Center (PATRIC), currently BV-BRC [20]. While this database includes clinical samples from diseased individuals, many samples are collected outside of clinical settings. Therefore, it is crucial to thoroughly inspect this data when drawing inferences from it.

### 1.3. Objectives of BacSPaD

BacSPaD (Bacterial Strains’ Pathogenicity Database) was developed to address these challenges by providing a rigorously curated database focused on the pathogenicity of bacterial strains. The integration of high-quality genomic data with detailed metadata from two reputable sources is supplemented by manual curation and scientific literature review to ensure the accuracy of pathogenicity annotations. By classifying bacterial genomes as HP or NHP based on consistent criteria, BacSPaD provides a valuable resource for researchers studying bacterial pathogenesis.

## 2. Materials and Methods

### 2.1. Data Acquisition

The data utilized in this work was primarily extracted from the BV-BRC database (Frederick, MD, USA) and supplemented with BioSample metadata from NCBI (Bethesda, MD, USA) [21], which provides comprehensive insights into specimen origins and phenotypic traits. We selectively sourced genomes from BV-BRC that were associated with a human host, marked as ‘good’ quality, fully sequenced (‘complete’), included both chromosomes and plasmids, and added to the database (‘insertion date’) after 1 January 2017, in order to balance data quality and volume. Then, the corresponding metadata from NCBI’s BioSample was retrieved via the Entrez system.

### 2.2. Data Pre-Processing and Integration

During pre-processing and integration, we meticulously identified and curated relevant fields from both databases. The final set of combined fields and their corresponding descriptions are shown in Supplementary Information: Table S1. This step involved conducting a detailed examination of metadata content, aligning common fields, and addressing discrepancies. For example, we resolved 8 instances where multiple NCBI BioSample entries corresponded to a single BV-BRC genome entry, possibly due to the submission of biological replicates or updated sample details. We assessed the metadata content to ensure that each genomic record was unique and removed the redundant entries. A total of 11,368 genomes to be annotated according to pathogenicity were retrieved after these pre-processing steps. The resulting enriched dataset laid the groundwork for our ensuing analysis and systematic annotation. A summary of the steps applied for the pre-processing and labeling phase, including the number of filtered and resulting genomes after each step, is detailed in Supplementary Information: Figure S1. We also assigned the taxonomy information for each genome from species to phylum based on NCBI taxonomy [22].

### 2.3. Quality Control

Following the annotation phase, a final high-quality selection step was performed. Labeled genomes were only kept if they showed over 90% completeness and less than 5% contamination, as confirmed using CheckM version v1.1.6 (Brisbane, Australia) [23]. Furthermore, we focused on primary pathogens, excluding entries associated with immunocompromised individuals. Exceptions were made if the species was listed in the FDA-ARGOS Database Wanted Organism List [24]. A list of the identified keywords associated with immunocompromised individuals used to guide this selection is shown in Supplementary Information: List S1. To further ensure sequence quality, 4 entries were removed as their genomes contained more than 20 contigs. Genomes associated with genetic manipulation in a research context were also excluded.

## 3. Results

### 3.1. Pathogenicity Annotation

Using a rule-based workflow, we systematically categorized bacterial strains as either HP or NHP, based on their association with infectious processes, or lack thereof. This process involved an iterative review of metadata, focusing on terms that effectively categorized genomes as HP or NHP based on their clinical context. These keywords could then not correspond to the most common ones found in the literature, whether specific to bacterial infections or broader medical terms. Instead, after an initial assessment using a broad set of keywords, we included only those that enhanced classification accuracy for both HP and NHP genomes within the context of our iterative procedure. Our goal was to create keyword lists tailored to this dataset, supporting manual review and reducing redundancy. We began the categorization process using keywords suggested by Naor-Hoffman et al. [10], based on metadata, to facilitate initial sorting. However, a significant number of genomes were misclassified, necessitating an iterative process of keyword inclusion and exclusion to enhance accuracy. This refinement led to the development of extensive keyword lists tailored for the metadata of the selected genomes, which guided the subsequent manual reviews. The final lists are detailed in Supplementary Information: List S2, S3, S4 and S5.

#### 3.1.1. HP Labeling Workflow

**Figure 1:**
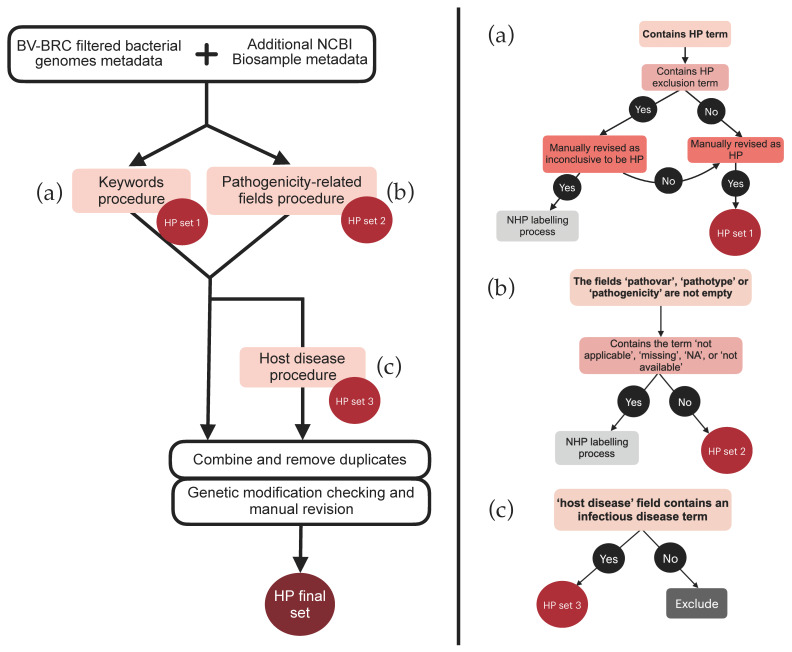
Outline of the rule-based criteria that guided the classification of genomes as pathogenic to humans (HP). The main processes applied to the expanded data are shown. These were based on the following: (**a**) HP keywords or their exclusion counterparts (HP set 1); (**b**) Inspection of metadata fields specifically indicative of their pathogenicity (HP set 2); (**c**) Association with an infectious disease in a specific metadata field (‘host disease’, HP set 3).

An initial selection was performed based on keywords related to virulence, disease manifestations, and distinctive HP features (Figure 1a). The final list of selected keywords was designated as ‘HP keywords’ (detailed in Supplementary Information: List S2). Conversely, keywords that usually correctly classified genomes as inconclusive were designated as ‘HP exclusion keywords’ (detailed in Supplementary Information: List S3). Genomes containing both an HP keyword and an HP exclusion keyword underwent a thorough review. If ambiguities remained, they were excluded from the HP category and reassessed under the NHP criteria. Ultimately, this process led to 4343 genomes being labeled as HP (HP set 1)

Additional genomes were labeled as HP based on metadata fields directly suggesting pathogenicity, such as ‘pathovar’, ‘pathotype’, or ‘pathogenicity’, unless these fields were empty or marked with ‘not applicable’, ‘missing’, or ‘not available’ (Figure 1b). In case they were, they would also be re-assessed under the NHP criteria. This process resulted in a total of 201 genomes being labeled as HP (HP set 2).

In order to further assess and incorporate genomes of HP strains that were not labeled with the previous processes, we took advantage of the ‘host disease’ metadata field, which specifies the disease affecting the host from which the sample was obtained. Manual inspection was facilitated by the smaller number of unlabeled genomes (Figure 1c). A total of 428 keywords related to documented infectious diseases were manually identified in this category (Supplementary Information: Table S2). Genomes associated with any of these keywords were added to the HP set after the removal of the modified strains. This process resulted in a total of 1046 genomes being categorized as HP (HP set 3). 

After combining the result of each of these steps, along with manual revision and the filtering of mutant strains, a final set of 5502 HP genomes was retrieved.

#### 3.1.2. NHP Labeling Workflow

**Figure 2:**
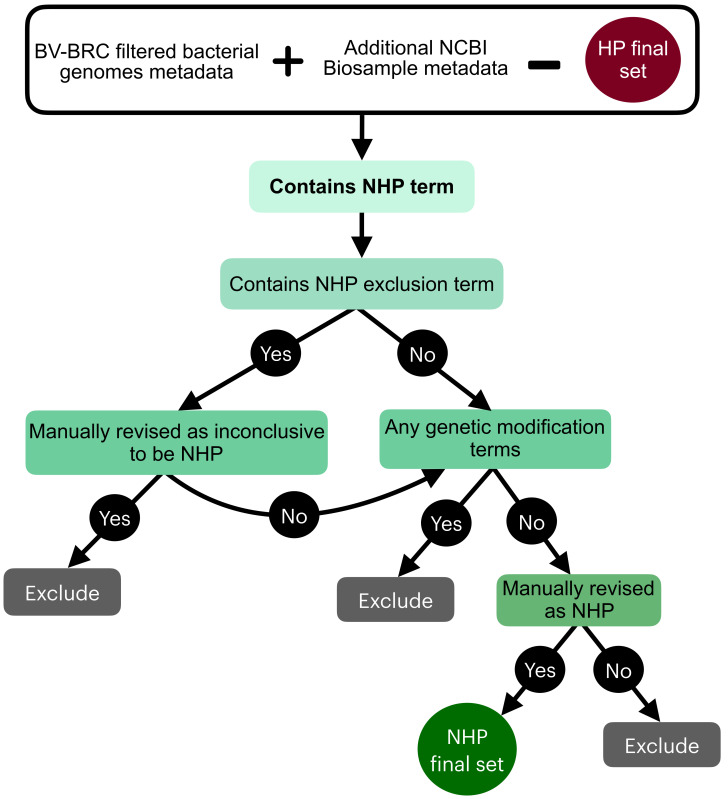
Outline of the rule-based criteria that guided the classification of genomes as non-pathogenic to humans (NHP).

The NHP labeling process was initiated by excluding genomes already identified as HP (Figure 2). Keywords associated with an NHP phenotype, designated as ‘NHP keywords’, were also derived from extensive metadata review to ensure no association with disease (Supplementary Information: List S4). Then, a verification of keywords that were found to help detect inconclusive genomes was also followed and these keywords were designated as ‘NHP exclusion keywords’ (Supplementary Information: List S5). Lastly, a similar scrutiny for strains associated with genetic modification was applied to these NHP genomes, but in this case no such strain was found in this condition. This process led to a final set of 490 genomes being definitively categorized as NHP.

#### 3.1.3. Manual Curation

The manual review involved a detailed examination of each genome’s classification, considering both the metadata and the latest scientific literature when necessary. Genomes with conflicting information—where metadata suggested a potentially NHP phenotype but was inconclusive, and literature indicated HP outcomes, or vice versa—were marked as inconclusive and were excluded from the dataset. This rigorous manual curation process ensured the reliability of our automated methods and the integrity of the final database.

### 3.2. Case Studies of HP and Inconclusive Genomes

Table 1 provides detailed examples of genomes categorized either as HP or inconclusive. For each genome, the table lists the most relevant metadata influencing their classification. The first two examples highlight scenarios where metadata contained both HP and exclusion keywords, necessitating a nuanced manual review to confirm their classification. For *Streptococcus pyogenes* strain M75, although healthy volunteers are mentioned, researchers successfully infected them using this strain [25]. Similarly, for *Neisseria meningitidis* strain S4, despite its species being described primarily as an obligate commensal, the metadata also states its “ability to cause septicemic disease and meningitis”, and that this strain in particular is an invasive strain.

In the last two rows of this table, we show examples from the set of genomes which contained an HP keyword and no HP exclusion keyword but were considered inconclusive after manual revision. For *Citrobacter koseri* strain MPUCK001, there was an association with atopic dermatitis, which, despite containing two HP terms (the ‘disease’ and the suffix ‘-itis’) is not an infectious disease. For *Pseudomonas putida* strain 15420352, which was isolated from a host with a pulmonary infection, the sample was taken from urine, and no information was given to substantiate an infection of the urinary tract.

### 3.3. Database Overview and Analysis

#### 3.3.1. General Statistics and Distribution

**Figure 3:**
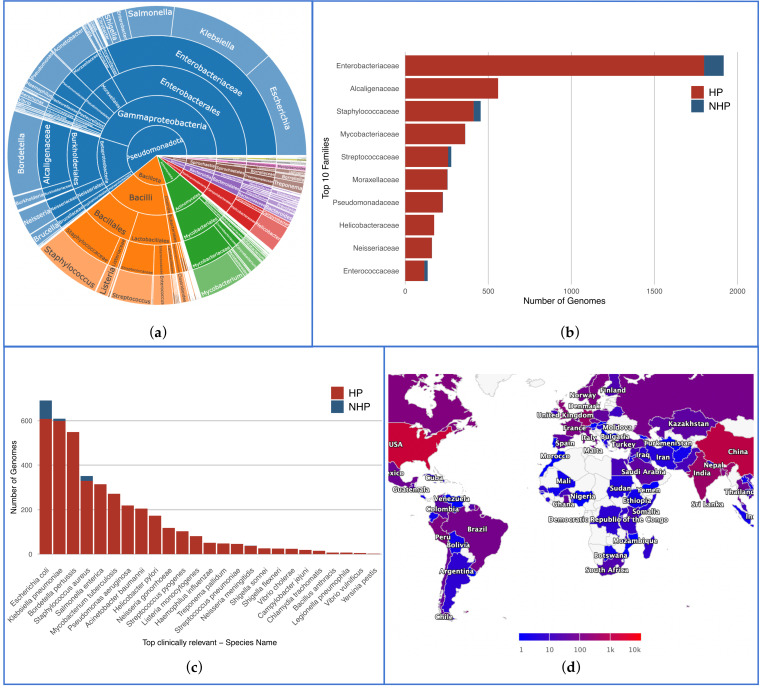
Key statistics from the database. (**a**) Distribution of various taxa, with each phylum assigned a different color; (**b**) Distribution of HP and NHP strains across the top 10 most frequent bacterial families; (**c**) Prevalence of HP and NHP strains across all species present in FDA-ARGOS Wanted Organism List; (**d**) Preview of the mapping figure showing the global distribution of bacterial strains by isolation country. The color legend ranges from 1 to 10 k, though the current maximum value is around 4 k, to accommodate future data.

We could annotate 5992 complete and high-quality genomes according to their pathogenicity—5502 as HP (92%), and 490 as NHP (8%). This database encompasses a broad spectrum of bacterial taxa, including 532 species across 193 genera, 96 families, 53 orders, 26 classes, and 12 phyla. The main taxa and their proportions are illustrated in Figure 3a. An interactive visualization of this figure may also be found in the web interface, enabling the visualization of all taxa. Figure 3b illustrates the distribution of the 10 most prevalent families, highlighting Enterobacteriaceae as the most frequent family, with 1799 HP and 118 NHP genomes. Accordingly, this family predominantly consists of clinically significant organisms such as Escherichia coli, Klebsiella, and Enterobacter. The second most frequent family is Alcaligenaceae (559 HP, 0 NHP), primarily due to the high prevalence of pathogenic strains of Bordetella pertussis, the agent responsible for whooping cough.

Other notable families include Mycobacteriaceae, Staphylococcaceae, and Streptococcaceae, underscoring their clinical significance with a significant representation of HP genomes.

We also validated the comprehensive nature of our database against the FDA-ARGOS Database Wanted Organism List, and verified that there were at least two genomes per species for the priority pathogens. Figure 3c displays the distribution of labeled genomes across all species featured in the list, with a minimum of 118 genomes for the top 10 species classified as HP. Specifically, the database includes 116 NHP genomes for this priority pathogens list: 83 *Escherichia coli*, 10 *Klebsiella pneumoniae*, 22 *Staphylococcus aureus*, and 1 *Neisseria meningitidis*.

Figure 3d presents a preview of the global map depicting the distribution of bacterial strains in the database based on their country of isolation. The color gradients represent the number of isolated strains, with darker shades indicating a higher number. Countries with extensive public health surveillance and research infrastructure show higher numbers of isolated strains.

#### 3.3.2. Virulence Factor Analysis

The analysis of virulence factors plays a pivotal role in identifying potential targets for drug development and assessing the risk of disease outbreaks. Although NHP strains can also harbor virulence genes, HP genomes are expected to contain a higher number of these factors. For a focused analysis, we selected a representative subset of 1484 genomes from clinically relevant species, maintaining an HP to NHP ratio of approximately 11:1, consistent with the overall database distribution. The selected genomes were aligned against experimentally verified virulence factors from the Virulence Factor Database [26] using Abricate v1.0.1 [27], which uses BLAST and a subject coverage threshold of 80%. A total of 2257 virulence factors were retrieved. Then, a chi-squared test with Yates’ correction was conducted to evaluate the association between the presence of virulence factors and HP classification. The analysis confirmed a statistically significant association (X-squared = 5.523, df = 1, *p*-value = 0.019), with an odds ratio of 1.26, suggesting a moderate positive correlation between the presence of virulence factors and HP classification. These identified virulence factors are also accessible through the web interface for further analysis.

#### 3.3.3. Database Structure

To support ongoing and future research endeavors, we have developed a web interface, which can be accessed freely at https://bacspad.altrabio.com/ (accessed on 6 August 2024).

- Data: Integrated dataset with pathogenicity annotation for each strain. Users can perform queries by any keyword across any field, as well as field-specific searches. Detailed descriptions of each metadata field are available in Supplementary Information: Table S1. Users may download selected genomes or retrieve them in batch along with various other data files, such as proteomes and protein families, from the BV-BRC FTP site at https://www.bv-brc.org/docs/quick_references/ftp.html (accessed on 6 August 2024). To facilitate the search for strains associated with a specific disease or isolation source category/subcategory, a categorization of diseases and isolation sources was also performed and the obtained fields added to this data. These were designated, respectively, as ‘disease category’, ‘disease subcategory’, and ‘isolation source’.
- Dashboard: This section features a range of statistical visualizations, including the top 10 and 50 species, the top 12 families, a location distribution map according to the country of isolation, and interactive visualizations of taxonomy, isolation sources, and disease categories with respective subcategories.
- Molecular Biology: This section includes visualizations on the distributions for plasmids and contigs counts, genome lengths in base pairs (‘bp’), GC content percentage, and protein-coding sequences (‘PATRIC CDS’).
- Virulence Factors: Virulence factor information for the most prevalent clinical species, including the gene name; the frequency at which it is found in HP strains; the frequency at which it is found in NHP strains; a list of the BV-BRC genome IDs in which it is found; the species names; and the corresponding number of strains, species, genera, and families.
- About: Summary of the utility of BacSPaD for microbiology research.

## 4. Discussion

Infectious diseases are a leading cause of illness and mortality globally. A key challenge in studying bacterial infection mechanisms and developing predictive models has been the absence of a comprehensive database that categorizes bacterial strains by their pathogenic potential in humans. The quality of data used is crucial for the reliability of these models. Without such a resource, previous studies have often relied on automated keyword matching or broad assumptions about bacterial isolation sources [7,9,10]. However, and as illustrated with the Case Studies in Section 3.2, this method often overlooks the subtleties in complex biological data. To address these limitations and provide a curated foundation resource, BacSPaD employs a rigorous manual curation process informed by the scientific literature. The NHP labeling of genomes from species usually regarded as HP, such as *Escherichia coli* and *Klebsiella pneumoniae*, highlights the necessity for nuanced, strain-level pathogenicity classification. Existing resources, such as BacDive [14] and gcPathogen [15], are not primarily designed for pathogenicity classifications. BacDive provides some bacterial pathogenicity information but lacks clear strain-level classifications and criteria, focusing only on HP classifications and excluding NHP ones. Similarly, gcPathogen provides only species-level pathogenicity classifications and also lacks NHP annotations. BacSPaD offers comprehensive strain-specific pathogenicity labeling for complete bacterial genomes, containing both HP and NHP annotations. This granular approach allows for more accurate representation of the variability in pathogenic potential within a species, a nuance that is missed in databases such as BV-BRC [20] or the Integrated Microbial Genomes database [13]. Moreover, by assessing important disease keywords from the ‘host disease’ metadata field, we were able to significantly increase the number of effectively labeled genomes. The further manual categorization of this field may also be of utility, mainly for researchers examining the disease associations of microbes and their specific pathogenic potential under varying health conditions. Finally, the exclusion of genomes associated with genetic modifications is a crucial step that has generally not been addressed in previous studies and current resources. This step ensures that our database reflects the natural dynamics of bacterial infections, allowing for more accurate computational studies and prediction tools of bacterial pathogenicity. The results are not confounded by artificial genetic changes that may alter virulence properties. Thus, BacSPaD provides a unique and valuable resource for enhancing predictive models of bacterial pathogenicity. For vaccine development, BacSPaD can aid in identifying conserved antigens prevalent in HP strains but less common in NHP strains. These antigens are key targets for broad-spectrum vaccines, capable of triggering an immune response against various bacterial species, even at the strain level. By eliciting an immune response, these vaccines help reduce the risk of infection and the development of resistance. Therefore, this database aligns with recent genomics-based vaccine advancements, highlighting the role of comprehensive resources in identifying effective vaccine targets. Integrating BacSPaD with omics technologies can significantly improve public health interventions and our ability to manage evolving bacterial infections.

Importantly, the virulence factor analysis revealed a statistically significant correlation between the number of virulence factors and pathogenic classification. Yet, the moderate odds ratio of 1.26 indicates that the predictive power of known virulence factors is limited. This finding underscores the need for a more integrative and comprehensive approach to understanding bacterial pathogenicity, which BacSPaD aims to facilitate. While the Virulence Factor Database [26] used for this analysis focuses on cataloging known virulence factors, BacSPaD provides a curated set of pathogenicity-labeled genomes that can be used to discover novel determinants of pathogenicity beyond currently known virulence factors. In addition, by including plasmids, BacSPaD also has the potential to enhance our understanding of these genetic elements. Plasmids play a critical role in bacterial pathogenicity by often carrying genes responsible for virulence factors and antibiotic resistance. This is particularly relevant in *Escherichia coli* and *Klebsiella pneumoniae*, where plasmid-encoded genes can result in severe, hard-to-treat infections, especially in clinical settings with prevalent multidrug-resistant strains [28,29]. Furthermore, the availability of identified virulence factors and associated genomes in the web interface is highly beneficial for researchers. This accessibility allows for further analysis and cross-referencing, providing a valuable resource for studying bacterial pathogenicity and developing targeted interventions.

However, our database is not without limitations. The genetic basis of pathogenicity was not assessed during the annotation process, and the inclusion of antimicrobial resistance data could enrich the database’s utility. The binary classification system—labeling genomes simply as HP or NHP—may not fully reflect the nuanced spectrum of bacterial pathogenicity. Future developments should consider a more sophisticated categorization system and ensure regular updates to the database to incorporate new genomes and re-evaluate classifications based on new research findings.

BacSPaD constitutes a comprehensive resource that covers a wide range of bacterial strains, offering flexibility and opportunities for cross-referencing. To minimize genomic redundancy, sequence comparison analyses, such as Average Nucleotide Identity (ANI) [30] or Mash [31], should be conducted as a preliminary step when selecting datasets from BacSPaD. These approaches help eliminate redundant entries, enabling a focus on unique HP and NHP features, thereby enhancing the precision and relevance of research findings.

In conclusion, our database presents a robust and comprehensive integrated resource of bacterial pathogenicity at the strain level. Future research will benefit from using it to assess global and specific determinants of bacterial pathogenicity, in order to further enrich our understanding of this complex field. Researchers may uncover patterns and develop prediction tools more effectively. This advancement may, in turn, significantly impact public health efforts in mitigating the problem of infectious diseases.

## Figures and Tables

**Table 1:** Examples of genomes labeled as HP and as inconclusive. Relevant metadata fields that influenced their classification are highlighted, with HP keywords marked by an asterisk (*) and HP exclusion keywords indicated by double asterisks (**). Some of the indicated HP keywords are not necessarily included in the final list of keywords but were important for the manual revision (e.g., ‘invasive’).

Species Name	Genome Name	Relevant Metadata Field(s) and Content	Label
*Streptococcus pyogenes*	*Streptococcus pyogenes* strain M75	Comments: “…modern controlled human infection * model, with the aim of safely and successfully causing pharyngitis * in healthy ** adult volunteers”	HP
*Neisseria meningitidis*	*Neisseria meningitidis* strain S4	Comments: “…ability to cause septicaemic disease * and meningitis * (…) meningococcus is primarily an obligate commensal ** of the human nasopharynx, and it is unclear why the bacterium has evolved exquisite mechanisms to avoid host immunity (…) genome of S4, an invasive * strain of *Neisseria meningitidis*”.	HP
*Citrobacter koseri*	*Citrobacter koseri* strain MPUCK001	Isolation source: “The skin surface of human (disease *: atopic dermatitis *) neck”	Inconclusive (after manual revision)
*Pseudomonas putida*	*Pseudomonas putida* strain 15420352	Isolation source: “urine”; host health: “pulmonary infection *”	Inconclusive (after manual revision)

## Data Availability

The original dataset from this study is available for free download in CSV format and can be accessed through the BacSPaD web interface at https://bacspad.altrabio.com (accessed on 6 August 2024), or directly via Zenodo under the DOI: https://doi.org/10.5281/zenodo.13235446. Additionally, the source code utilized for the labeling procedure and the construction of the web interface is hosted on our GitHub repository at https://github.com/ribeirosara/BacSPaD (accessed on 6 August 2024). Both the dataset and the source code are provided under the Creative Commons Attribution-NonCommercial-ShareAlike 4.0 International License (CC BY-NC-SA 4.0), promoting open and collaborative scientific endeavors.

## Supplementary Materials

The following supporting information can be downloaded at https://www.mdpi.com/article/10.3390/pathogens13080672/s1: Table S1: BacSPaD genome metadata fields and their corresponding descriptions; Figure S1: Summary of the steps applied for the pre-processing phase, including filtration and refinement of bacterial genomes data and the respective labeling phase; List S1: Final list of keywords associated with immunocompromised hosts; List S2: Final list of HP keywords; List S3: Final list of HP exclusion keywords; Table S2: Final list of infectious disease keywords and their frequency; List S4: Final set of NHP keywords; List S5: Final set of NHP exclusion keywords.
