# Supplementary material for: BacSPaD: A Robust Bacterial Strains’ Pathogenicity Resource Based on Integrated and Curated Genomic Metadata
Source: Pathogens. 2024 Aug 9;13(8):672. doi: 10.3390/pathogens13080672 (PMC11357117; doi:10.3390/pathogens13080672)
Supplement: Supplementary file 1 [file pathogens-13-00672-s001.zip › pathogens-3119657-supplementary.pdf]

Table S1: BacSPaD genome metadata fields and their corresponding description.

Metadata fields	Description
pathogenicity label	Labeling according to pathogenicity - either nonpathogenic to humans (NHP) or pathogenic to humans (HP).
genome id	Genome ID from Bacterial and Viral Bioinformatics Resource Center (BV-BRC) database.
genome name	Genome name.
strain	Strain name according to National Center for Biotechnology Information (NCBI) taxonomy.
species	Species name according to NCBI taxonomy.
genus	Genus name according to NCBI taxonomy.
family	Family name according to NCBI taxonomy.
order	Order name according to NCBI taxonomy.
class	Class name according to NCBI taxonomy.
phylum	Phylum name according to NCBI taxonomy.
biosample accession	BioSample accession number ID from NCBI.
taxon id	Taxon ID from NCBI taxonomy.
serovar	Taxonomy below subspecies; a variant which is usually based on its antigenic properties. Same as serotype (https://www.ncbi.nlm.nih.gov/biosample/docs/attributes/).
biovar	Variant distinguished by its unique biochemical or physiological traits (https://www.ncbi.nlm.nih.gov/biosample/docs/attributes/).
pathovar	Taxonomy below subspecies; a variety usually based on its pathogenic properties. Sometimes used as equivalent to subspecies. (https://www.ncbi.nlm.nih.gov/biosample/docs/attributes/)
mlst	Genotypic identifier based on housekeeping gene sequences.
other typing	Strain typing or characterization methods beyond the standard approaches such as MLST (Multilocus Sequence Typing). Each 'genotype' followed by a number (e.g., genotype:1 or genotype:1903) denotes a unique genetic profile or pattern that has been identified in the microbial species under investigation.
culture collection	Reference to a deposited microbial strain in a repository, identified by a unique accession number.
type strain	Indication if it is a type strain ('yes' or ''). A type strain is a nomenclatural standard for a particular bacterial species, serving as a reference point for its definition and identification.
completion date	Date of project completion.

Metadata fields	Description
publication	Associated scientific publication identifier.
bioproject accession	Unique identifier to corresponding project in NCBI.
assembly accession	Unique identifier to corresponding genome assembly in NCBI. Refers to a specific version of a genome assembly submitted to a database like NCBI's GenBank.
genbank accessions	Unique identifier(s) of GenBank assembly/assemblies in NCBI.
refseq accessions	Unique identifiers assigned to sequences within the Reference Sequence (RefSeq) database. RefSeq sequences are curated by NCBI staff and collaborators.
sequencing centers	Sequencing center (e.g. University 'x', Hospital 'y').
sequencing platform	Sequencing platform (e.g. Illumina, PacBio).
sequencing depth	Average number of times each nucleotide in a genome is sequenced.
assembly method	Methodology used to assemble the genomic sequences.
chromosomes	Number of associated chromosomes.
plasmids	Number of associated plasmids.
contigs	Number of associated contigs.
genome length	Genome length measured in base pairs (bp).
gc content	Measure of the proportion of guanine (G) and cytosine (C) nucleotides in the DNA sequence, expressed as a percentage of the total nucleotide composition.
patric cds	Number of protein-coding sequences (CDS) annotated or sourced from PATRIC (previous version of BV-BRC).
refseq cds	Number of protein-coding sequences (CDS) annotated or sourced from RefSeq database.
isolation source	Corresponding origin of isolation. This attribute provides information about the ecological niche or source of the bacterial strain.
isolation comments	Additional notes or comments regarding the isolation of a specific bacterial strain.
collection date	Date on which a specific bacterial strain was collected or isolated from its source.
isolation country	Country associated with the biological sample isolation.
geographic location	Geographical descriptors associated with the biological sample isolation.
other environmental	Supplementary attribute to describe specific environmental conditions or contexts associated with the biological sample.
host gender	Host gender.
host age	Host age.
host health	Host health status or condition.

Metadata fields	Description
body sample site	Specific anatomical site or location from which the biological sample was collected.
other clinical	Additional clinical information or metadata associated with the biological sample.
antimicrobial resistance	This field shows genomes that have been specifically tested against certain antibiotics and the resulting phenotype from that test. Note that a genome can have multiple antibiotic phenotypes, such as being resistant to one drug and susceptible to another. Values in this field include 'Resistant','Susceptible' or 'Intermediate' (https://www.bv-brc.org/docs/quick_references/organisms_taxon/antimicrobial_resistance.html).
antimicrobial resistance evidence	Indicates the information source behind the AMR designation. Allowable values include 'Computational Prediction", 'Computational Method' , and 'AMR Panel" (https://www.bv-brc.org/docs/quick_references/organisms_taxon/antimicrobial_resistance.html)
gram stain bvbrc	Gram staining information ("positive" or "negative") sourced from BV-BRC.
cell shape	Cell shape information (e.g. Bacilli, Cocci).
motility	Motility information ("yes": motile, "no": non-motile).
temperature range	Indication on phenotype associated with range of temperature at which the organism is known to thrive, survive, or exhibit optimal growth (e.g. 'Mesophilic').
optimal temperature	Optimal temperature at which the organism is known to exhibit optimal growth.
oxygen requirement	Specific oxygen conditions a microorganism requires to survive; Values include 'Aerobic', 'Anaerobic', 'Facultative', or 'Microaerophilic'.
habitat	Natural or artificial habitat in which the bacteria resides or was found.
disease	Host disease.
comments	Supplementary information in form of comments providing further contextual details.
additional metadata	Supplementary metadata providing further contextual details.
env broad scale	Broad-scale environmental context (https://www.ncbi.nlm.nih.gov/biosample/docs/attributes/).
env local scale	Local-scale environmental context (https://www.ncbi.nlm.nih.gov/biosample/docs/attributes/).
env medium	Environmental medium/material. keywords describing the material displaced by the entity during sampling (https://www.ncbi.nlm.nih.gov/biosample/docs/attributes/).
isol growth condt	Description or URL indication of isolation and growth condition specifications (https://www.ncbi.nlm.nih.gov/biosample/docs/attributes/).
project name	A concise name that describes the overall project (https://www.ncbi.nlm.nih.gov/biosample/docs/attributes/).
pathogenicity details	Additional bacterial strain information on pathogenicity (e.g. 'commensal', or 'diphtheria-like symptoms').
host disease	Name of relevant disease, e.g. Salmonella gastroenteritis (https://www.ncbi.nlm.nih.gov/biosample/docs/attributes/).
host health state	Information regarding health state of the individual sampled at the time of sampling (https://www.ncbi.nlm.nih.gov/biosample/docs/attributes/).
host disease outcome	Final outcome of disease, e.g., death, chronic disease, recovery.

Metadata fields	Description
host description	Additional host information not included in other defined vocabulary fields (https://www.ncbi.nlm.nih.gov/biosample/docs/attributes/).
host disease stage	Stage of disease at the time of sampling (https://www.ncbi.nlm.nih.gov/biosample/docs/attributes/).
pathotype	Bacterial specific pathotype (e.g. *Escherichia coli* - STEC, UPEC) https://www.ncbi.nlm.nih.gov/biosample/docs/attributes/
subsource note	Subsource note. Further details about the origin, isolation method, or other relevant information regarding the sample used.
note	Additional note. This can include details about the source of the sequence, experimental conditions, characteristics of the organism, or any other relevant information.
description	Further details on isolation source or organism.
biotic relationship	Observed biotic relationship ([", 'free living', 'parasite', 'commensal', 'symbiont']) https://www.ncbi.nlm.nih.gov/biosample/docs/attributes/
biome	Major environment type(s) where sample was collected (https://www.ncbi.nlm.nih.gov/biosample/docs/attributes/).
host status	Information on host health status.
risk group	Risk group classification - based on their potential hazard to human health and the environment (species-level, ranges from 1 to 3; 3 representing the highest hazard).
Note on infection mode	Further details on infection mode.
checkm compl final	Genome completeness (%) according to CheckM tool v1.1.6.
checkm contam final	Genome contamination (%) according to CheckM tool v1.1.6.
disease category	Disease category (e.g. Respiratory diseases).
disease subcategory	Subcategory of the main disease category (e.g. Pneumonia). When the specific infectious disease name is not available, an associated keyword is given instead (e.g. Pertussis).
isolation source category	Isolation source category (e.g. Respiratory tract).
disease comb	Combination of the disease category and disease subcategory (e.g. Respiratory diseases - Pneumonia).

Figure S1: Summary of the steps applied for the pre-processing phase, including filtration and refinement of bacterial genomes data and respective labeling phase.

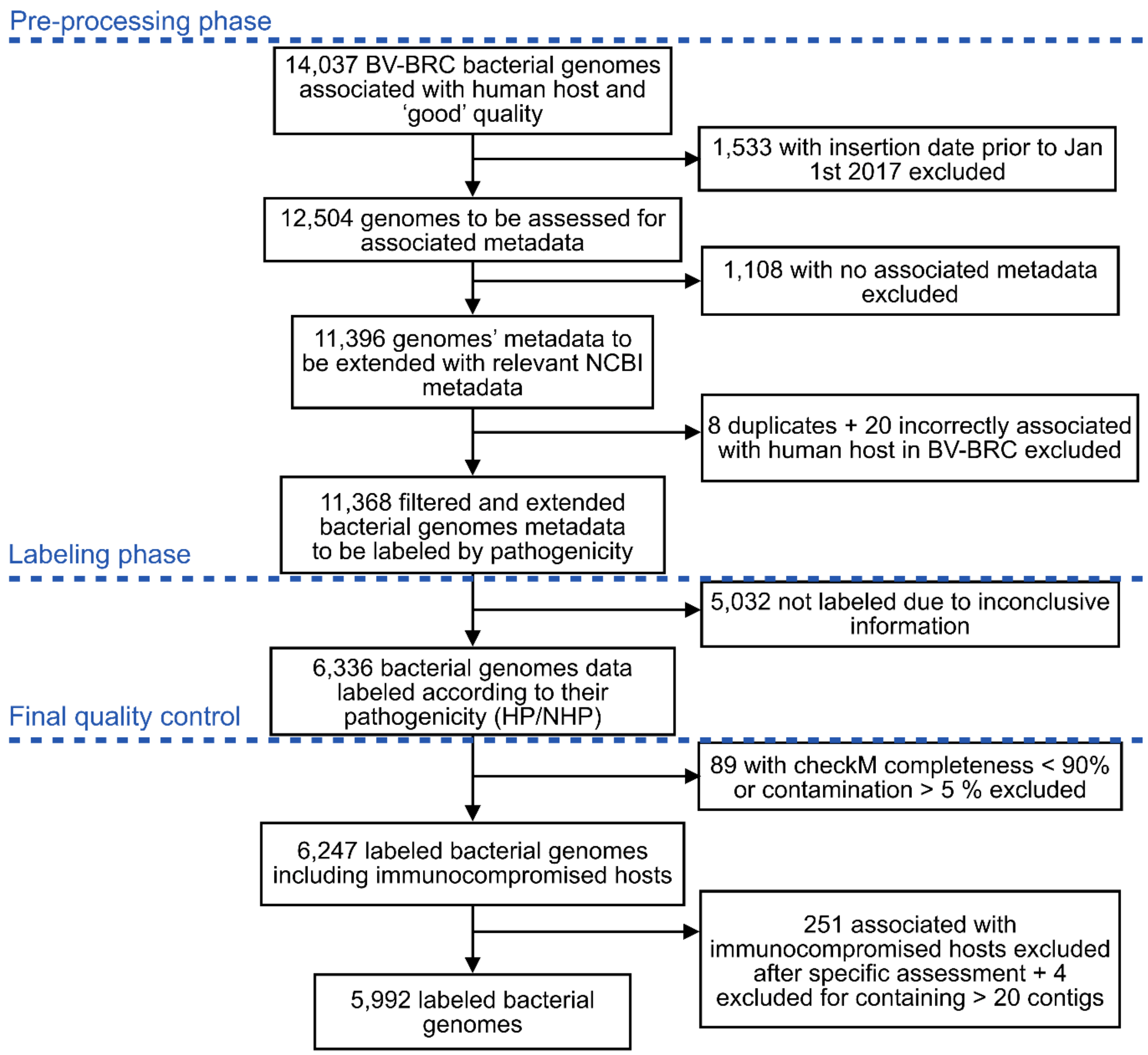

List S1: Final list of keywords associated with immunocompromised hosts

'immunocompromised', 'diabetes', 'cancer', 'HIV', 'AIDS', 'leukemia', 'carcinoma', 'Guillain', 'interleukin-12 receptor deficiency'.

List S2: Final list of HP keywords

'virulence', 'superbug', 'waterborne', 'foodborne', 'outbreak', 'infection', 'pathogen', 'water borne', 'food borne', keywords with suffix "-itis", 'poisoning', 'infectious', 'sepsis', 'infected', 'biofilm', 'purulent', 'pus', 'death', 'severe', 'diseased', 'pandemic',

‘epidemic’, ‘transmission’, ‘vector’, ‘toxin’, ‘toxic’, ‘clinical’, ‘biosafety level 2’, ‘hypervirulent’, ‘diarrhea’, ‘intensive’.

List S3: Final list of HP exclusion keywords

‘Healthy’, ‘probiotic’, ‘commensal’, ‘microbiome’, ‘microbiota’, ‘nutraceutical’, ‘normal’, ‘asymptomatic’, ‘naturally occurring’, ‘human-associated habitat’, ‘opportunistic’.

Table S2: Final list of infectious disease keywords and their frequency

Infectious disease or associated keyword	Frequency
bacteremia	199
tuberculosis	134
pneumonia	109
gonorrhoea	65
whooping cough	64
brucellosis	51
gastric ulcer	36
uti	36
gonorrhea	27
clostridium difficile	23
syphilis	23
compound ulcer	18
pertussis	17
listeriosis	16
shigellosis	13
tularemia	11
bacterial vaginosis	10

**Infectious disease or associated keyword**	**Frequency**
fever	9
melioidosis	9
septicemia	9
diphtheria	8
pneumoniae	8
typhoid fever	8
abscess	7
liver abscess	7
salmonellosis	7
bacteraemia	6
salmonella	6
bartonella quintana	5
cholera	5
duodenal ulcer	5
scarlet fever	5
septicaemia	5
actinomycetoma	4
febrile illness after i. scapularis tick-bite	4
hemolytic-uremic syndrome	4
human bacteremia with s. aureus	4
mycobacterium tuberculosis	4
bacterimia	3
botulism	3
dental caries	3

**Infectious disease or associated keyword**	**Frequency**
human bacteremia with s. aureus (mrsa)	3
rheumatic fever	3
salmonella gastroenteritis	3
septic shock	3
skin sore / abscess / burns / iv site	3
urine infection	3
bejel	2
glanders	2
gonorrhoeae	2
lemierre's syndrome	2
leptospirosis	2
nosocomial infection	2
pnuemonia	2
rocky mountain spotted fever	2
ventilator-associated pneumonia	2
yaws	2
a. baumannii bacteremia	1
abdominal abscess	1
abscess neck	1
acinetobacter infections	1
acute leukemia, pneumonia	1
anthrax	1
atrophic gastic	1
bacteremia without focus	1

**Infectious disease or associated keyword**	**Frequency**
bacterial pneumonia	1
bilateral pneumonia	1
blood	1
blood stream	1
brain congestion	1
bronchial granuloma	1
bsi	1
cepacia syndrome	1
copd	1
corneal ulcer	1
dysentery	1
eczema herpeticum	1
endemic syphilis	1
enteric fever	1
erysipelas	1
erysipeloid	1
facial abscessus	1
far east scarlet-like fever	1
fatal septicaemia	1
fever of unknown origin	1
gastric ulcers	1
gbs infection	1
gluteal abscess	1
healthcare-associated pneumonia	1

**Infectious disease or associated keyword**	**Frequency**
hip abscess	1
hiv; cat scratch disease	1
infant botulism	1
infection	1
infenction	1
interstitial pneumonia	1
liver abscessus	1
localized aggressive periodontitis	1
lung abscess	1
lyme borreliosis	1
mac pneumonia, hiv	1
mediastinal abscess	1
periapical abscess	1
perisplenic abscess	1
pneamonia	1
pneumococcal disease	1
pneumonia and bacteremia	1
pneumonia, bacterial	1
polysegmental community acquired pneumonia	1
refractory periapical abscess	1
rickettsiosis	1
secondary tuberculosis	1
septic shock, multiple organ failure	1
skin abscess	1

Infectious disease or associated keyword	Frequency
soft tissue infection	1
spastic tetraplegia. chronic right-sided pneumonia, exacerbation.	1
stec infection	1
urinary tract infection	1
yersiniosis	1

List S4: Final list of NHP keywords

‘Healthy’, ‘probiotic’, ‘Commensal’, ‘microbiome’, ‘microbiota’, ‘symbiotic’, ‘nutraceutical’, “normal’, ‘commercial’, ‘flora’.

List S5: Final list of NHP exclusion keywords

‘patient’, ‘abscess’, ‘wound’, ‘bacteremia’, ‘pneumonia’, ‘ICU ‘, ‘disease’, ‘contaminated’, ‘symptom’, ‘clinic’.
